# Investigation of the presence and persistence of bacteria in seawater and oysters from an aquaculture farm in Rehoboth Bay, Delaware

**DOI:** 10.1128/spectrum.03054-24

**Published:** 2025-04-10

**Authors:** Kelvin F. Ofori, Ali Parsaeimehr, Gulnihal Ozbay

**Affiliations:** 1 Food Science and Biotechnology Program, Department of Human Ecology, College of Agriculture, Science and Technology, Delaware State University 557726, Dover, Delaware, USA; 2 Department of Agriculture and Natural Resources, College of Agriculture, Science and Technology, Delaware State University 222333 , Dover, Delaware, USA; University of Minnesota Twin Cities, St. Paul, Minnesota, USA

**Keywords:** aquaculture, bacteria, detection, oysters, contamination, microbial safety

## Abstract

The filter-feeding nature of oysters, anthropogenic activities, and increasing agriculture in Delaware compromise the microbial safety of Eastern oysters from local aquaculture farms. From July to October 2023, we evaluated the presence and persistence of eight bacteria in seawater and oysters produced from off-bottom and bottom cultures at Sally Cove, an aquaculture farm within Rehoboth Bay in Delaware. A control site within Sally Cove, which was without oyster cultures, was also included in the study. Seawater temperature, salinity, pH, and dissolved oxygen were measured *in situ* during sampling. Molecular confirmation with PCR and qPCR showed that *Vibrio parahaemolyticus*, Shiga-toxin-producing *Escherichia coli*, *Salmonella enterica*, *Staphylococcus aureus*, *Pseudomonas aeruginosa*, and *Clostridium* spp. were present and persisted in seawater and oyster samples from both cultures at Sally Cove and in off-bottom and bottom seawater samples from the control site throughout the study. *Shigella* spp. and *Listeria monocytogenes* were consistently found in seawater and oyster samples from July to September. However, *Shigella* spp. was only detected in samples from the bottom cultures, whereas *L. monocytogenes* was undetectable in all samples from both cultures in October. The observed temperature, salinity, pH, and dissolved oxygen levels across the study period were in the range of 15.30–29.67°C, 29.33–31.87 ppt, 7.25–7.95, and 3.79–8.10 mg/L, respectively, and comparable with the conditions suitable for the growth and survival of these bacteria. These findings suggest that consuming raw oysters from Sally Cove poses contamination risks from several bacteria, especially in the summer months.

**IMPORTANCE:**

Although studies have evaluated bacterial contamination in seawater and oysters within the Delaware Inland Bays and nearby areas, the focus has primarily been on *Vibrio* species. However, other bacteria have been found in seawater and seafood at various locations and could potentially occur in oysters produced from aquaculture farms within the Delaware Inland Bays. Sally Cove is an oyster aquaculture farm that produces Eastern oysters (*Crassostrea virginica*) for consumption in Delaware using both off-bottom and bottom culturing methods. The risk of bacterial contamination from consuming raw oysters from this farm is unknown. This paper shows the presence and persistence of several bacteria, including those associated with waste, in seawater and oysters at the farm. The findings can inform consumers about the contamination risks from consuming raw oysters produced at the farm.

## INTRODUCTION

Oysters are among the seafood choices for consumers in the USA. They are rich in zinc and omega-3 fatty acids and are great sources of proteins (1). The demand for oysters in the USA is increasing, with a rise in total supply from 55,627,000 pounds in 2019 to 78,845,000 pounds in 2022 (2). Ecologically, they play important roles in marine ecosystems by contributing to shoreline protection, improving water quality and carbon sequestration, and providing reef habitats for other aquatic organisms (3). As natural biofilters, oysters can filter approximately 5–25 liters of water per hour (4). Through this filter-feeding nature, oysters can accumulate foodborne pathogens, coastal pollutants, and marine toxins in their digestive glands, posing significant health risks if consumed raw (5, 6).

In the USA, there are about 48 million infections, 128,000 hospitalizations, and 3,000 deaths annually from foodborne diseases (7). Seafood has been a significant vector for foodborne outbreaks. The Centers for Science in Public Interest (CSPI) reported that more than 500 solved outbreaks and approximately 5,000 associated illnesses between 2003 and 2012 in the USA were linked to seafood (8). Bacteria are implicated in most seafood-borne infections and accounted for 76.1% of all seafood-related outbreaks in the USA from 1973 to 2006 (9). Studies have reported the presence of *Vibrio parahaemolyticus* (10–12), Shiga-toxin-producing *Escherichia coli* (13, 14), *Salmonella enterica* (15, 16), *Shigella* spp. (17, 18), *Staphylococcus aureus* (19, 20), *Listeria monocytogenes* (21, 22), *Pseudomonas aeruginosa* (23, 24), and *Clostridium* spp. (25, 26) in seawater, oysters, and seafood in the USA and other locations. The occurrence of these bacterial pathogens in seawater and seafood is influenced by abiotic factors such as water temperature, salinity, pH, dissolved oxygen, and rainfall (1, 10, 12, 18). Although post-harvest processes like depuration have been developed to remove these contaminants, they are not always effective (1). Therefore, continuous monitoring of bacterial pathogens in seafood is important for ensuring seafood safety and protecting public health (16).

In 2020, the expansion of shellfish aquaculture, including oyster farming, was promoted in the USA to help close a seafood trade deficit estimated at over $14 billion. Consequently, permits were issued to shellfish growers for shellfish production in coastal areas (6). Delaware Inland Bays and other coastal areas within the Atlantic Coast in the USA are used extensively for Eastern oysters (*Crassostrea virginica*) gardening programs (27). Sally Cove in Rehoboth Bay is one of the oyster aquaculture farms along the Atlantic coast of Delaware that produces oysters through off-bottom and bottom cultures for consumption in Delaware. The filter-feeding nature of oysters, anthropogenic activities, and increased agriculture in Delaware potentially compromise the microbial safety of oysters from this aquaculture farm (27). Most studies that evaluated bacterial contamination in marine environments and oysters in the Delaware Inland Bays have targeted *Vibrio* species (Table 1). Moreover, the risk of bacterial contamination from consuming raw oysters from this farm is unknown. The objective of this study was to evaluate the presence and persistence of several bacteria in seawater and oysters from both off-bottom and bottom cultures at Sally Cove across different months. A control site within Sally Cove that is without oyster cultures was included in this study. In this study, we (i) determined the presence and persistence of eight different bacteria in off-bottom and bottom seawater from Sally Cove and the control site, and in oysters collected from off-bottom and bottom cultures at Sally Cove, and (ii) assessed the physicochemical seawater quality parameters, including temperature, salinity, pH, and dissolved oxygen from July to October 2023. The findings will serve as a foundation for subsequent studies to evaluate the prevalence and concentration of the detectable bacteria at the targeted oyster farm.

**TABLE 1:** Summary of studies on the detection of bacterial pathogens in seawater and oysters within Delaware Inland Bays and nearby areas

Study	Bacteria	Sample	Location	Detection technique
Parveen et al. (11)	*V. parahaemolyticus*	Seawater and oysters	Chesapeake Bay	Enrichment and real-time PCR
Richards et al. (28)	*Shewanella* spp.	Seawater and oysters	Delaware Bay	Biochemical tests and sequencing
Main et al. (29)	*Vibrio* spp.	Seawater	Delaware Inland Bays	Quantitative PCR
Davis et al. (30)	*V. parahaemolyticus*	Seawater	Chesapeake Bay	Quantitative PCR
Ozbay et al. (27)	*Vibrio* spp.	Seawater and Oysters	Delaware Inland Bays	COPP assay
Ozbay et al. (1)	*Vibrio* spp., total aerobic bacteria, and fecal coliforms	Oysters	Delaware Inland Bays	COPP assay and MPN
Almuhaideb et al. (10)	*V. parahaemolyticus*	Seawater and oysters	Delaware Bay	PCR
Parveen et al. (12)	*V. parahaemolyticus* and *V. vulnificus*	Seawater and oysters	Delaware and Chesapeake Bays	Direct plating and MPN-PCR
Rosales et al. (31)	*V. parahaemolyticus* and *V. vulnificus*	Seawater	Rehoboth Bay	Real-time PCR

## RESULTS

### Molecular detection and persistence of bacteria in seawater and oysters

**Fig 1:**
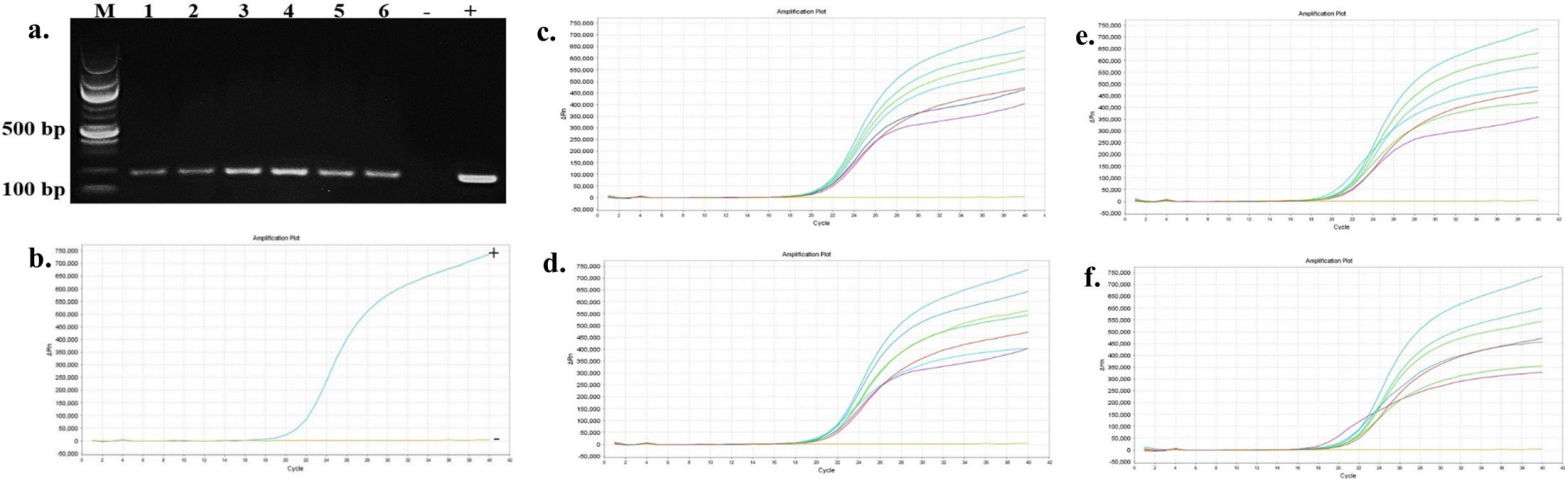
Molecular confirmation of *V. parahaemolyticus* in seawater and oyster samples across the study period. (a) is a representation of the PCR amplification showing the presence of *V. parahaemolyticus* in samples as observed during each month; (b) demonstrates the amplification curve of positive and negative controls with qPCR; and (c-f) demonstrate the amplification curves showing the presence of *V. parahaemolyticus* in seawater and oyster samples during (c) July, (d) August, (e) September, and (f) October months with qPCR. Note: 100 bp ladder (M) was used; 1: Bottom seawater from Sally Cove; 2: Off-bottom seawater from Sally Cove; 3: Bottom seawater from control site; 4: Off-bottom seawater from control site; 5: Bottom-cultured oysters from Sally Cove; 6: Off-bottom-cultured oysters from Sally Cove; -: Negative control; +: Positive control.

**Fig 2:**
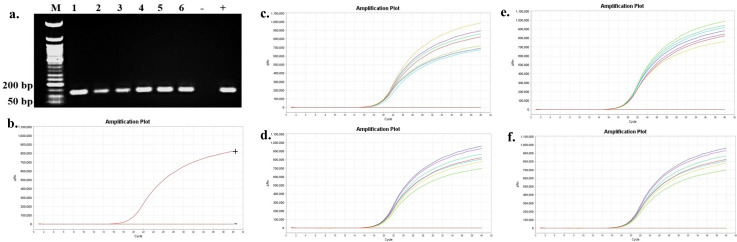
Molecular confirmation of Shiga-toxin-producing *E. coli* in seawater and oyster samples across the study period. (a) is a representation of the PCR amplification showing the presence of Shiga-toxin-producing *E. coli* in samples as observed during each month; (b) demonstrates the amplification curve of positive and negative controls with qPCR; and (c–f) demonstrates the amplification curves showing the presence of Shiga-toxin-producing *E. coli* in seawater and oyster samples during (c) July, (d) August, (e) September, and (f) October months with qPCR. Note: 50 bp ladder (M) was used; 1: Bottom seawater from Sally Cove; 2: Off-bottom seawater from Sally Cove; 3: Bottom seawater from control site; 4: Off-bottom seawater from control site; 5: Bottom-cultured oysters from Sally Cove; 6: Off-bottom-cultured oysters from Sally Cove; -: Negative control; +: Positive control.

**Fig 3:**
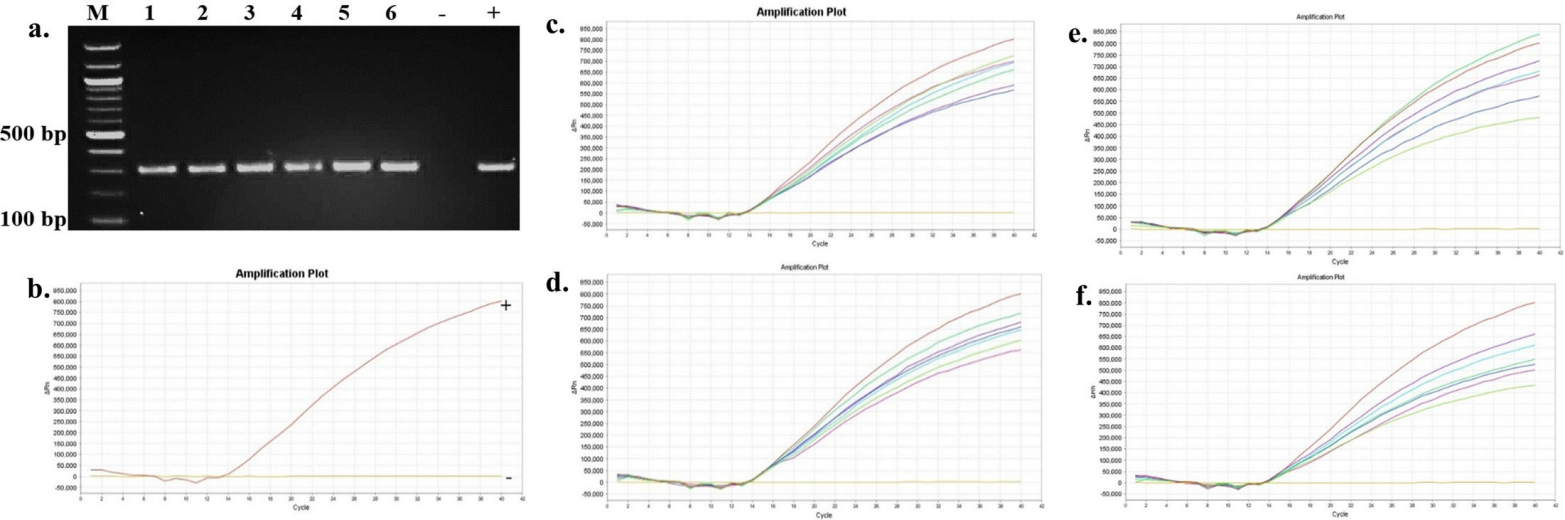
Molecular confirmation of *S. enterica* in seawater and oyster samples across the study period. (a) is a representation of the PCR amplification showing the presence of *S. enterica* in samples as observed during each month; (b) demonstrates the amplification curve of positive and negative controls with qPCR; and (c–f) demonstrate the amplification curves showing the presence of *S. enterica* in seawater and oyster samples during (c) July, (d) August, (e) September, and (f) October months with qPCR. Note: 100 bp ladder (M) was used; 1: Bottom seawater from Sally Cove; 2: Off-bottom seawater from Sally Cove; 3: Bottom seawater from control site; 4: Off-bottom seawater from control site; 5: Bottom-cultured oysters from Sally Cove; 6: Off-bottom-cultured oysters from Sally Cove; -: Negative control; +: Positive control.

**Fig 4:**
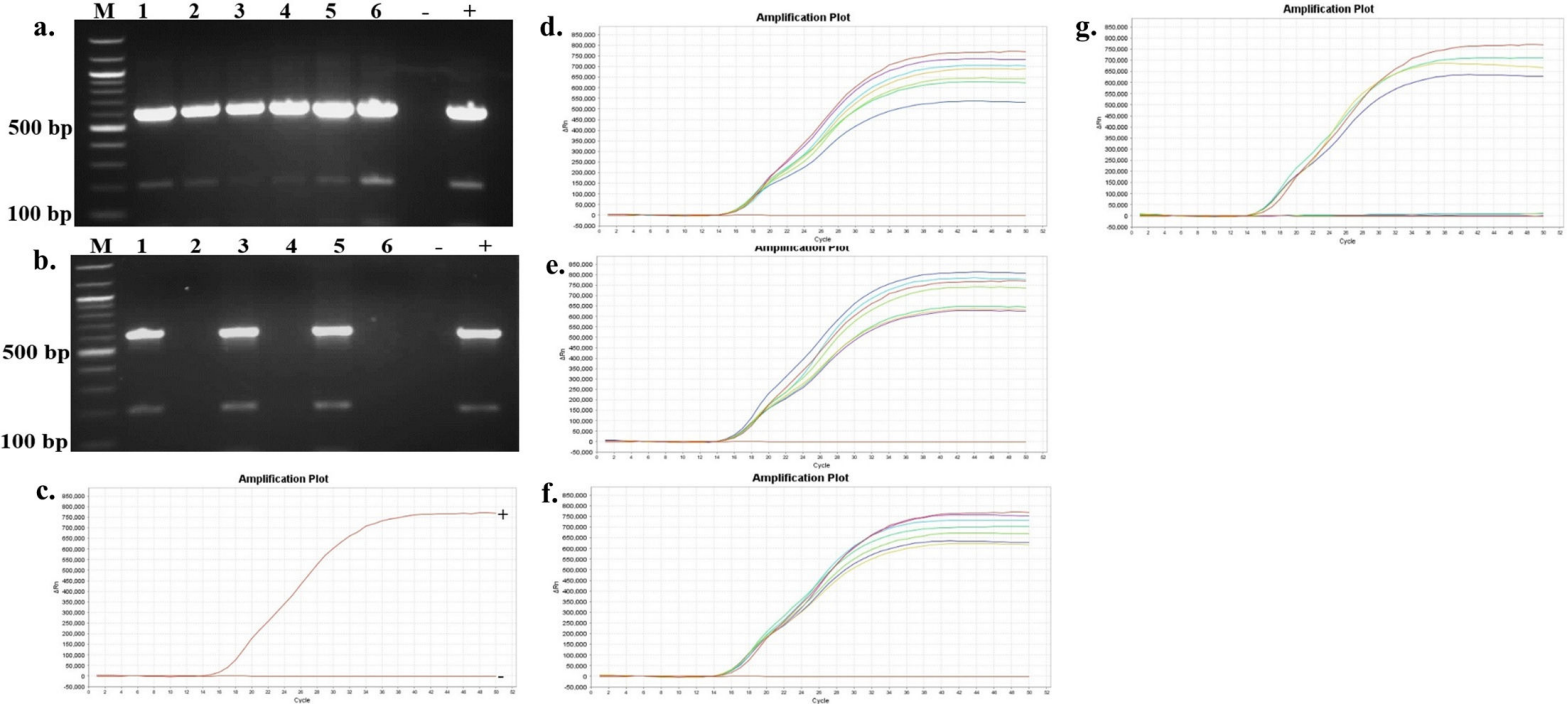
Molecular confirmation of *Shigella* spp. in seawater and oyster samples across the study period. (a and b) is a representation of the PCR amplification showing (a) the presence of *Shigella* spp. in samples as observed during July, August, and September months, and (b) the presence of *Shigella* spp. in only bottom seawater and oyster samples from bottom cultures during October; (c) demonstrates the amplification curve of positive and negative controls with qPCR; and (d–g) demonstrates the amplification curves showing the presence of *Shigella* spp. in seawater and oyster samples during (d) July, (e) August, (f) September months, and (g) the presence of *Shigella* spp. in only bottom seawater and oyster samples from bottom cultures during October with qPCR. Note: 100 bp ladder (M) was used; 1: Bottom seawater from Sally Cove; 2: Off-bottom seawater from Sally Cove; 3: Bottom seawater from control site; 4: Off-bottom seawater from control site; 5: Bottom-cultured oysters from Sally Cove; 6: Off-bottom-cultured oysters from Sally Cove; -: Negative control; +: Positive control.

The presence and persistence of several bacteria in the seawater and oysters from off-bottom and bottom cultures at the oyster aquaculture farm (Sally Cove) and in off-bottom and bottom seawater at its control site across four sampling months (July to October 2023) were evaluated in this study. The bacteria were initially detected using selective media, followed by confirmation of presumptive colonies with PCR and further validation using quantitative PCR (qPCR). *V. parahaemolyticus* was detected in both the off-bottom and bottom seawater samples from Sally Cove and the control site from July to October months through molecular confirmation of the thermolabile hemolysin (*tlh*) gene (Fig. 1). Similarly, it persisted in both off-bottom and bottom-cultured oysters from Sally Cove across the entire study period. Molecular confirmation of the Shiga-toxin-producing (*stx_2_*) gene showed that Shiga-toxin-producing *E. coli* (STEC) was present in both off-bottom and bottom seawater samples from Sally Cove and the control site during each sampling month of the study period (Fig. 2). It was also detected in the off-bottom and bottom-cultured oysters from Sally Cove throughout the study. Similar findings were observed for *S. enterica,* which persisted in the off-bottom and bottom seawater samples from Sally Cove and the control site across the study period (Fig. 3). The same observations were made for off-bottom and bottom-cultured oysters from Sally Cove, where molecular confirmation of the invasion protein (*invA*) gene showed the persistence of *S. enterica* throughout the study. The molecular confirmation of the invasion plasmid antigen-encoding (*ipaH*) gene showed the presence of *Shigella* spp. in off-bottom and bottom seawater samples from Sally Cove and the control site during July, August, and September months (Fig. 4). The same outcomes were observed for off-bottom and bottom-cultured oysters from Sally Cove within the same period. However, *Shigella spp*. was detected only in bottom seawater samples from Sally Cove and the control site, as well as in bottom-cultured oysters from Sally Cove in October. It was undetectable in off-bottom seawater samples from both sites and in off-bottom-cultured oysters from Sally Cove in October.

**Fig 5:**
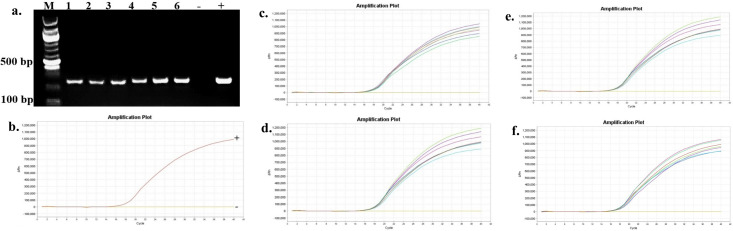
Molecular confirmation of *S. aureus* in seawater and oyster samples across the study period. (a) is a representation of the PCR amplification showing the presence of *S. aureus* in samples as observed during each month; (b) demonstrates the amplification curve of positive and negative controls with qPCR; and (c–f) demonstrate the amplification curves showing the presence of *S. aureus* in seawater and oyster samples during (c) July, (d) August, (e) September, and (f) October months with qPCR. Note: 100 bp ladder was used; 1: Bottom seawater from Sally Cove; 2: Off-bottom seawater from Sally Cove; 3: Bottom seawater from control site; 4: Off-bottom seawater from control site; 5: Bottom-cultured oysters from Sally Cove; 6: Off-bottom-cultured oysters from Sally Cove; -: Negative control; +: Positive control.

**Fig 6:**
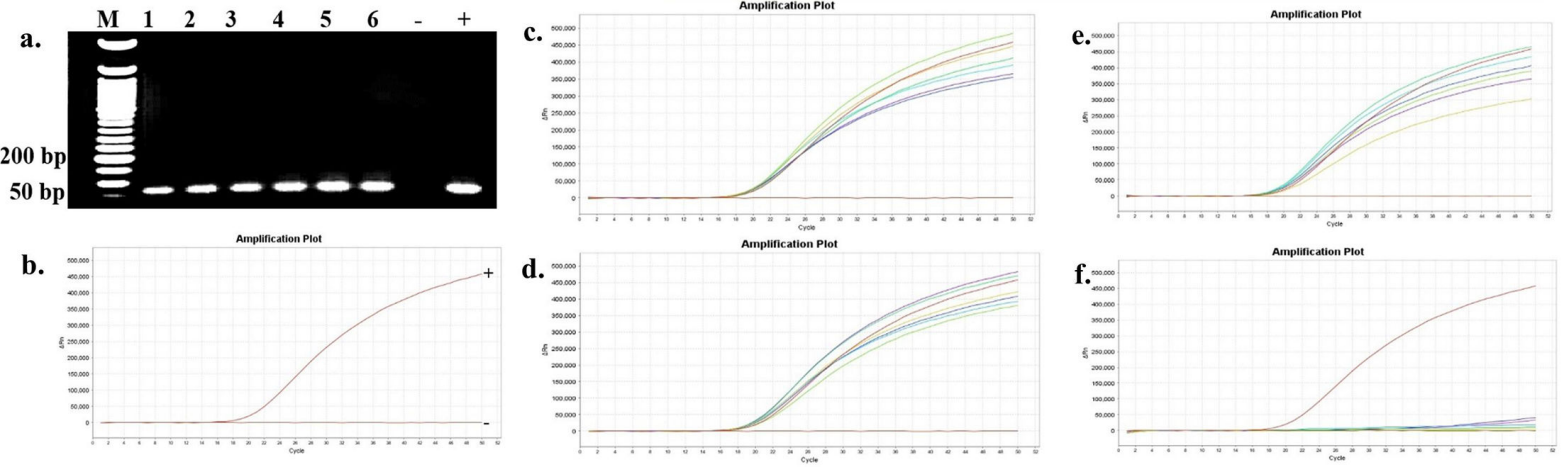
Molecular confirmation of *L. monocytogenes* in seawater and oyster samples across the study period. (a) is a representation of the PCR amplification showing the presence of *L. monocytogenes* in samples as observed during July, August, and September months; (b) demonstrates the amplification curve of positive and negative controls with qPCR; (c–e) demonstrate the amplification curves showing the presence of *L. monocytogenes* in seawater and oyster samples during (c) July, (d) August, and (e) September months; and (f) demonstrates the amplification plot showing the absence of *L. monocytogenes* in samples during October with qPCR. Note: 50 bp ladder (M) was used; 1: Bottom seawater from Sally Cove; 2: Off-bottom seawater from Sally Cove; 3: Bottom seawater from control site; 4: Off-bottom seawater from control site; 5: Bottom-cultured oysters at Sally Cove; 6: Off-bottom-cultured oysters at Sally Cove; -: Negative control; +: Positive control.

**Fig 7:**
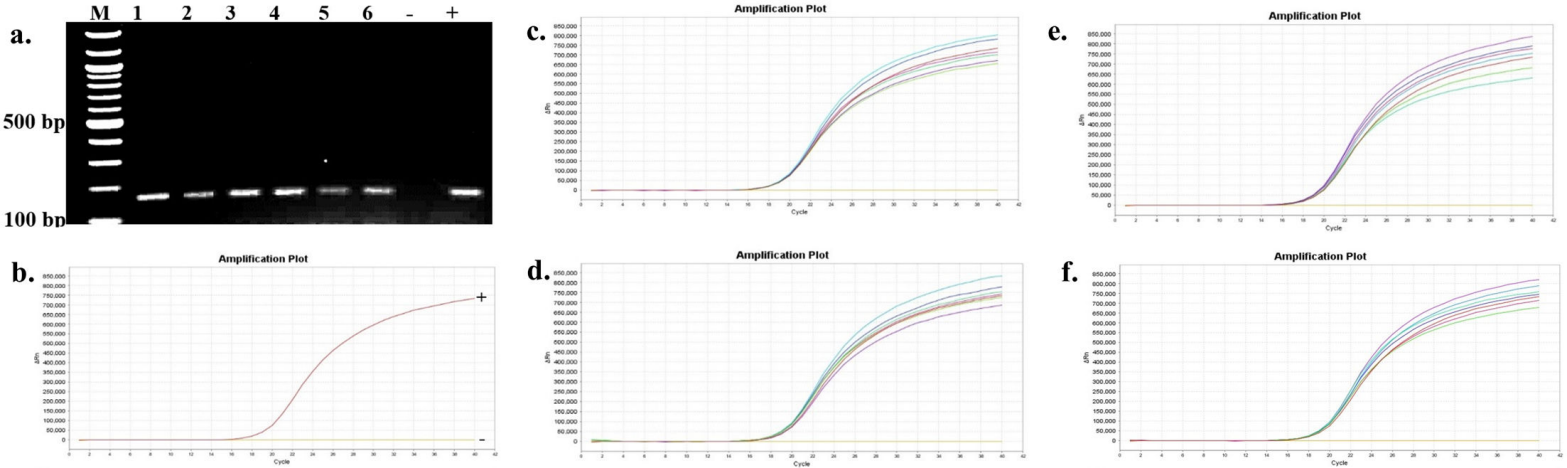
Molecular confirmation of *P. aeruginosa* in seawater and oyster samples across the study period. (a) is a representation of the PCR amplification showing the presence of P. aeruginosa in samples as observed during each month; (b) demonstrates the amplification curve of positive and negative controls with qPCR; and (c–f) demonstrate the amplification curves showing the presence of P. aeruginosa in seawater and oyster samples during (c) July, (d) August, (e) September, and (f) October months with qPCR. Note: 100 bp ladder (M) was used; 1: Bottom seawater from Sally Cove; 2: Off-bottom seawater from Sally Cove; 3: Bottom seawater from control site; 4: Off-bottom seawater from control site; 5: Bottom-cultured oysters from Sally Cove; 6: Off-bottom-cultured oysters from Sally Cove; -: Negative control; +: Positive control.

**Fig 8:**
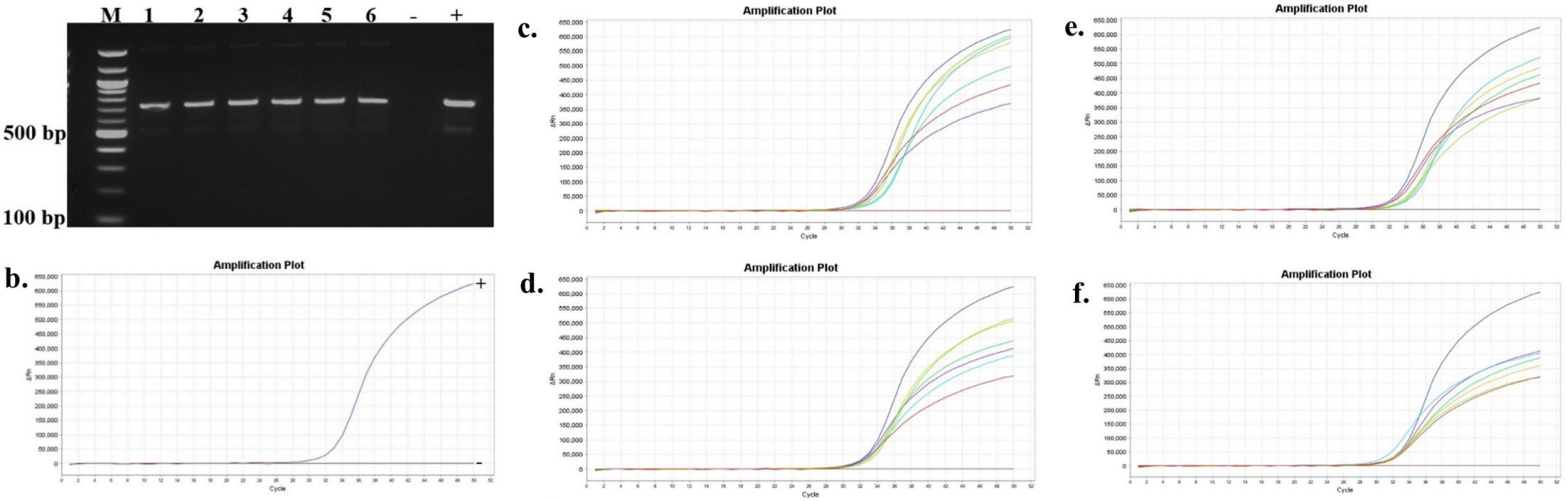
Molecular confirmation of *Clostridium spp*. in seawater and oyster samples across the study period. (a) is a representation of the PCR amplification showing the presence of *Clostridium spp*. in samples as observed during each month; (b) demonstrates the amplification curve of positive and negative controls with qPCR; and (c–f) demonstrates the amplification curves showing the presence of *Clostridium spp*. in seawater and oyster samples during (c) July, (d) August, (e) September, and (f) October with qPCR. Note: M: 100 bp ladder (M) was used; 1: Bottom seawater from Sally Cove; 2: Off-bottom seawater from Sally Cove; 3: Bottom seawater from control site; 4: Off-bottom seawater from control site; 5: Bottom-cultured oysters from Sally Cove; 6: Off-bottom-cultured oysters from Sally Cove; -: Negative control; +: Positive control.

*S. aureus* was detected in off-bottom and bottom seawater samples from Sally Cove and the control site during each sampling month of the study period through the molecular confirmation of the thermostable nuclease (*nucA*) gene (Fig. 5). The off-bottom and bottom-cultured oysters from Sally Cove also displayed the persistence of *S. aureus* across the study period. *L. monocytogenes* was detected in off-bottom and bottom seawater samples from Sally Cove and the control site during July, August, and September months through molecular confirmation of its *16S rRNA* gene (Fig. 6). It was also found in the off-bottom and bottom-cultured oysters from Sally Cove within the same period. However, it was undetectable in all seawater and oyster samples from both Sally Cove and the control site in October. Both off-bottom and bottom seawater samples from Sally Cove and the control site showed the presence of *P. aeruginosa* across the study period (Fig. 7). It also persisted in the off-bottom and bottom-cultured oysters from Sally Cove throughout the study period. Similarly, molecular confirmation of *16 s rRNA* of *Clostridium* spp. showed that it persisted in off-bottom and bottom seawater samples from Sally Cove and the control site from July through October (Fig. 8). The same observations were made for off-bottom and bottom-cultured oysters from Sally Cove. In summary, all the bacteria that were detected and persistent in the off-bottom and bottom seawater samples from Sally Cove were also found to be present and persistent in their corresponding oysters throughout this study (Table S1). Moreover, all the bacteria that were detected and persistent in the off-bottom and bottom seawater samples from Sally Cove were also detectable and persistent in the off-bottom and bottom seawater samples from the control site. Additionally, all eight targeted bacteria were detected in seawater and oyster samples from both cultures at Sally Cove during July, August, and September months. However, in October, six of the targeted bacteria were detectable in seawater and oyster samples from the off-bottom cultures, whereas seven were detectable in seawater and oyster samples from the bottom cultures.

### Physicochemical water quality parameters of study sites

**Fig 9:**
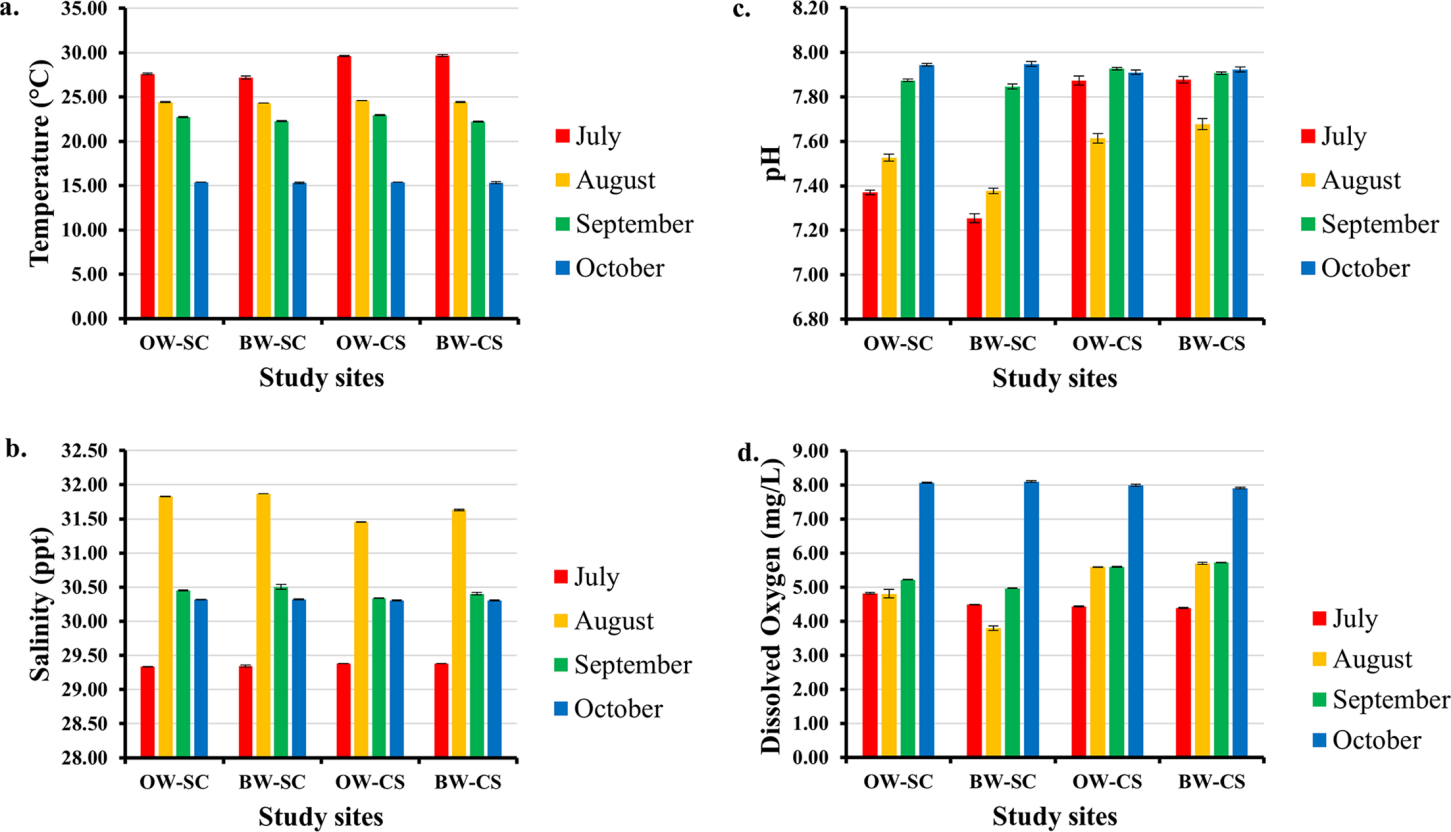
Average physicochemical water quality parameters of off-bottom and bottom seawater at study sites across the study period. The figure demonstrates the levels of (a) temperature, (b) salinity, (c) pH, and (d) dissolved oxygen. Abbreviations: OW-SC, Off-bottom seawater at Sally Cove; BW-SC, Bottom seawater at Sally Cove; OW-CS, Off-bottom seawater at the control site; BW-CS, Bottom seawater at the control site.

Abiotic water quality parameters were assessed *in situ* within the off-bottom and bottom seawater at Sally Cove and the control site from July to October (Fig. 9). Over the entire study period, seawater temperatures ranged from the lowest of 15.30°C in October in the bottom seawater at Sally Cove to the peak of 29.67°C in July in the bottom seawater at the control site. Seawater temperatures decreased gradually in both off-bottom and bottom seawater at Sally Cove and the control site from July to October. Salinity levels ranged from 29.33 to 31.87 ppt, with the lowest observed in off-bottom seawater at Sally Cove in July and the highest in bottom seawater at Sally Cove in August. Generally, salinity levels were highest in August in both off-bottom and bottom seawater at Sally Cove and the control site. The pH levels of the seawater in this study were in the range of 7.25–7.95, with both the lowest and highest observed in bottom seawater at Sally Cove in July and October, respectively. The minimum dissolved oxygen (DO) value of 3.79 mg/L and maximum value of 8.10 mg/L were both observed in the bottom seawater at Sally Cove in August and October, respectively.

## DISCUSSION

The physicochemical water quality parameters of the oyster aquaculture farm (Sally Cove) and its control site (no oyster cultures) were assessed from July through October during the sampling of seawater and oysters. These months were chosen for this study because summer and warmer seasons have been associated with a rise in occurrences and abundance of pathogens in seafood (10, 27). Expectedly, seawater temperatures decreased gradually at Sally Cove and the control site from July to October, with the lowest temperatures observed in October. July, August, and early September months are within the summer season when environmental temperatures are generally higher, whereas there is a drop in environmental temperatures during the fall season in October. The temperature range of 15.30–29.67°C observed in this study is comparable with the ranges of 14.63–28°C and 10.59–27.05°C reported by Almuhaideb et al. (10) in Delaware Bay and Scro et al. (32) in Wellfleet Bay, respectively. In their studies, the peak seawater temperature was observed in August and gradually decreased by October, similar to the observations in this study. Seawater salinity affects the survival and persistence of bacteria in marine environments and seafood (20, 30, 33). A similar salinity range was reported by Scro et al. (32) in Wellfleet Bay (27.29–32.62 ppt), whereas a much lower range was reported by Parveen et al. (11) in Chesapeake Bay (4.7–14.1 ppt), likely due to differences in geographical location and study duration. The pH and DO have also been shown to affect the occurrence of bacteria such as *V. parahaemolyticus*, *Staphylococcus* spp., *Pseudomonas* spp., and fecal indicator bacteria in marine environments and seafood (1, 11, 12, 18). The observed pH (7.25–7.95) and DO (3.79–8.10 mg/L) range in the study are consistent with those reported within Rehoboth Bay and its surrounding bays (Delaware and Chesapeake bays) (10–12, 31).

*V. parahaemolyticus* is indigenous to coastal environments and accounts for the majority of *Vibrio* infections (5). In the USA, an estimated 52,000 cases of foodborne vibriosis occur each year, typically from the consumption of raw or undercooked bivalve shellfish (34). The presence and persistence of *V. parahaemolyticus* in seawater and oyster samples throughout this study are consistent with those reported by other studies within surrounding sites and bays (5, 10, 12). *V. parahaemolyticus* is halophilic and occurs naturally in marine waters, which could account for its persistence in seawater and oysters throughout this study. The growth of *V. parahaemolyticus* is favored at temperatures above 15°C, an alkaline pH (7.9–8.6), and 3% NaCl (33). Moreover, surface temperatures of 25.67 ± 2°C, salinity range of 27.87 ± 3 ppt, and pH range of 7.96 ± 0.1 were found to be the most suitable conditions for the prevalence of *V. parahaemolyticus* in marine environments (35). The temperature (15.30–29.67°C), salinity (29.33–31.87 ppt), and pH (7.25–7.95) range observed in this study are comparable with these optimum ranges and could explain why *V. parahaemolyticus* persisted in the seawater and oysters throughout the study.

*E. coli* is found in the intestines of humans and animals. It is used as a bioindicator for fecal contamination and verifying water and fish quality (14). In the USA, STEC O157 leads to an estimated 97,000 illnesses, 3,270 hospitalizations, and 30 deaths each year (36). Based on past research, this study is the first to report on the presence of STEC within Rehoboth Bay. Similar findings on the detection and persistence of STEC in seawater, oysters, and other shellfish were reported in shellfish-harvesting sites on the French coast (37, 38), coastal shores of Lake Timsah in Egypt (14), and coastal environments in Morocco (13). The detection and persistence of STEC in seawater and oysters from both cultures at Sally Cove in this study indicate potential fecal contamination (39). This contamination could originate from runoff from nearby residential and agricultural areas or increasing footfall at Rehoboth Beach during the summer. *E. coli* can survive and grow at temperatures as low as 10°C and in a wide range of 23–40°C (40, 41), pH range of 4.4–9.0 (42), and in aerated waters (43). The temperature, pH, and DO levels observed in this study were consistent with these conditions, making it suitable for the persistence of STEC in the seawater and oyster samples throughout this study, despite changes in the physicochemical water quality parameters. Similar observations were made by Florini et al. (44), who found no seasonal variations in *E. coli* counts in water and oysters from oyster beds at an estuary in the UK. Boufafa et al. (18) also reported comparable temperature, pH, and DO ranges and detected and enumerated *E. coli* in seawater (at least three sites) and mussels (four sites) from July to October within the Gulf of Annaba.

*S. enterica* is mainly introduced into aquatic environments through fecal materials from livestock, birds, and feral animals, as well as from proximity to human sewage effluents (16). In the USA, *Salmonella* is one of the primary causes of foodborne illnesses and the leading cause of foodborne-related hospitalizations and deaths. It is responsible for approximately 1.35 million infections annually (45). Authorities have established a zero-tolerance policy for *Salmonella* due to its seafood-associated illnesses (46). The detection and persistence of *S. enterica* in the seawater and oyster samples throughout this study correspond with findings from other studies (15–17, 47). *Salmonella* can survive and multiply in a wide range of temperatures (7–48°C) and pH (4.05–9.50) (48). This ability may account for its persistence in the seawater and oyster samples throughout this study, as the observed temperature and pH levels were within the suitable ranges for its survival. Moreover, *Salmonella* has been shown to tolerate and withstand high salinity levels (16, 49), which could explain its persistence throughout the salty seawater in this study.

Foodborne *Shigella* infections typically result from poor sanitation and hygiene (50). In the USA, *Shigella* causes an estimated 450,000 infections annually, and its antimicrobial-resistant infections lead to an economic loss of $93 million in direct medical costs (51). The co-presence of STEC, *S. enterica*, and *Shigella* spp., which are human-enteric bacteria, in seawater and oysters from Sally Cove in this study strongly suggests contamination of the seawater at Sally Cove with human and animal waste. Unlike seawater and oyster samples from the bottom cultures, where *Shigella* spp. persisted throughout this study, it was present from July to September but undetectable in October in the seawater and oyster samples from the off-bottom cultures. Contrasting findings for the off-bottom cultures were observed by Jeamsripong et al. (17), where *Shigella* spp. was positive in estuarine water and oysters sampled in October. The discrepancies could be due to the sampling depth, as their study sampled surface seawater from 0.3 to 0.5 m below the water surface, compared with a lower depth of 0.1 m in this study. Marine sediment can host several bacteria and has been attributed to higher concentrations of *Vibrio* spp. in bottom seawater and oysters than in off-bottom seawater and oysters in previous studies (32, 52). This could explain the persistence of *Shigella* spp. throughout this study of seawater and oyster samples from bottom cultures, which were closer to the sediment floor, compared with those from the off-bottom cultures.

*S. aureus* is often found in human nasal, throat, and skin flora and has the ability to adapt to varying environmental conditions (19). It produces staphylococcal enterotoxins that are primarily associated with foodborne poisoning (19). The presence and persistence of *S. aureus* in seawater and oyster samples throughout this study are consistent with findings observed in coastal waters, oysters, and other shellfish (19, 20, 53). Curiel-Ayala et al. (53) attributed the higher concentration of *S. aureus* found in seawater on the Mexican Pacific Coast at 3 p.m. in both rainy and dry seasons to the peak tourist presence at that time. This could account for the persistence of *S. aureus* throughout this study, as Rehoboth Beach experiences high human populations during the summer. *S. aureus* can grow and survive in a wide range of temperatures (7.0–48.5°C), pH (4.0–10.0), and salinity (≤25% NaCl) (54). Moreover, at a DO range of 5.0–12.6 mg/L, *S. aureus* was detected in seawater and mussel samples (18). The temperature, salinity, pH, and DO levels observed in this study were comparable with these conditions and might have provided favorable conditions for the persistence of *S. aureus* in the seawater and oyster samples throughout this study.

*L. monocytogenes* accounted for 30% of all 2,400 recalls of seafood products from October 2002 through March 2022 in the USA (55). In the USA, *Listeria* infections rank third among the leading causes of foodborne mortalities. They affect an estimated 1,600 people and cause 260 deaths each year (56). Due to its high mortality rate, a zero tolerance for *L. monocytogenes* in food has been included in regulations by the U.S. Food and Drug Administration (FDA) (57). El-Shenawy & El-Shenawy (21) found a positive association between fecal contaminants and the presence of *L. monocytogenes* in coastal environments, noting that both bacteria are commonly found in sewage. Similarly, there was a co-presence and persistence of STEC and *L. monocytogenes* in seawater and oyster samples during the July to September months in this study. *L. monocytogenes* can survive and persist in a broad range of environments with temperatures ranging from –0.4 to 45°C, pH levels between 4.6 and 9.5, and salinity up to 20% (58). This adaptability may account for its persistence in seawater and oyster samples from July to September months in this study. Nevertheless, *L. monocytogenes* was undetectable in all samples in October in this study. Although *L. monocytogenes* is a facultative anaerobe, studies have shown that it exhibits improved growth and survival under low-oxygen and oxygen-restricted environments compared to oxygenated and high-oxygen environments, particularly in response to cold temperatures (59, 60). This could explain why *L. monocytogenes* was undetectable in seawater and oyster samples in October as temperatures decreased and dissolved oxygen levels increased.

*P. aeruginosa* is an opportunistic pathogen found in diverse environments such as soil, plants, and water (23, 24). In aquaculture, the presence of pathogenic *P. aeruginosa* can be used as an indicator of low water quality (24). Therefore, its presence and persistence in seawater and oyster samples throughout this study indicate that seawater at Sally Cove is of low quality and may not be suitable for oyster aquaculture. Several studies also reported the presence and persistence of *P. aeruginosa* in coastal environments, oysters, and other shellfish (23, 24, 61). *P. aeruginosa* has the tendency to grow at a wide range of temperatures (4–42°C), pH (4.5–9.0), and at high salinity (62, 63) and may account for its persistence in seawater and oyster samples throughout this study.

*Clostridium* is an anaerobic bacteria that forms spores, contributing to its persistence in a wide range of environments and its ability to withstand high temperatures (64). The detection and persistence of *Clostridium* spp. in the seawater and oysters sampled in each month in this study align with observations of previous studies (25, 26, 64). *Clostridium* spp. has been associated with sewage and found to be resilient in wastewater effluents, which consequently contaminate water and shellfish (25, 65, 66). Therefore, its persistence in seawater and oyster samples throughout this study indicates potential contamination of seawater at Sally Cove with domestic sewage. The quality of oysters from aquaculture farms is greatly influenced by the quality of culture waters (67) and may account for the simultaneous presence and persistence of all the targeted bacteria in seawater and their corresponding oysters from both cultures at Sally Cove in this study. Barr et al. (68) reported that the physiological activities and filtration behavior of Eastern oysters increased during warmer months at Barnegat, Delaware, and Rehoboth Bays. This increased filtration behavior may lead to rapid accumulation of bacterial pathogens, which may explain the detection of all the targeted bacteria in the oysters from both off-bottom and bottom cultures during the July, August, and September months in this study.

In summary, this study detected several bacteria that pose contamination risks from consuming raw oysters produced at Sally Cove, an aquaculture farm within Rehoboth Bay. *V. parahaemolyticus* and *P. aeruginosa* were consistently found in off-bottom and bottom seawater samples from Sally Cove and the control site, and in oysters from both cultures at Sally Cove across this study. Additionally, STEC, *S. enterica*, *S. aureus*, and *Clostridium* spp. were present and persistent in seawater and oyster samples from both cultures at the study sites throughout this study, indicating potential contamination of human, animal, and domestic waste at the aquaculture farm. *Shigella* spp. and *L. monocytogenes* persisted in seawater and oyster samples from both cultures at the study sites from July to September, but *Shigella* spp. was only detected in seawater and oyster samples from the bottom cultures in October, whereas *L. monocytogenes* was undetectable in all samples from both cultures in October. The observed temperature, salinity, pH, and dissolved oxygen levels in this study were comparable with the suitable ranges for the growth and survival of these bacteria. The detection and persistence of these bacteria in both seawater and oysters at the study location indicate potential concerns regarding seawater quality and highlight the need for further investigation into contamination risks, particularly in the summer season, to ensure the safety of consuming raw oysters from the study location. Moreover, future studies could focus on developing rapid assays such as the colorimetric and visual chromogenic chip assays that could be used by shellfish farmers in Rehoboth Bay for on-site detection and screening of these bacteria to enhance seafood safety monitoring.

## MATERIALS AND METHODS

### Description of the study sites and sample collection

**Fig 10:**
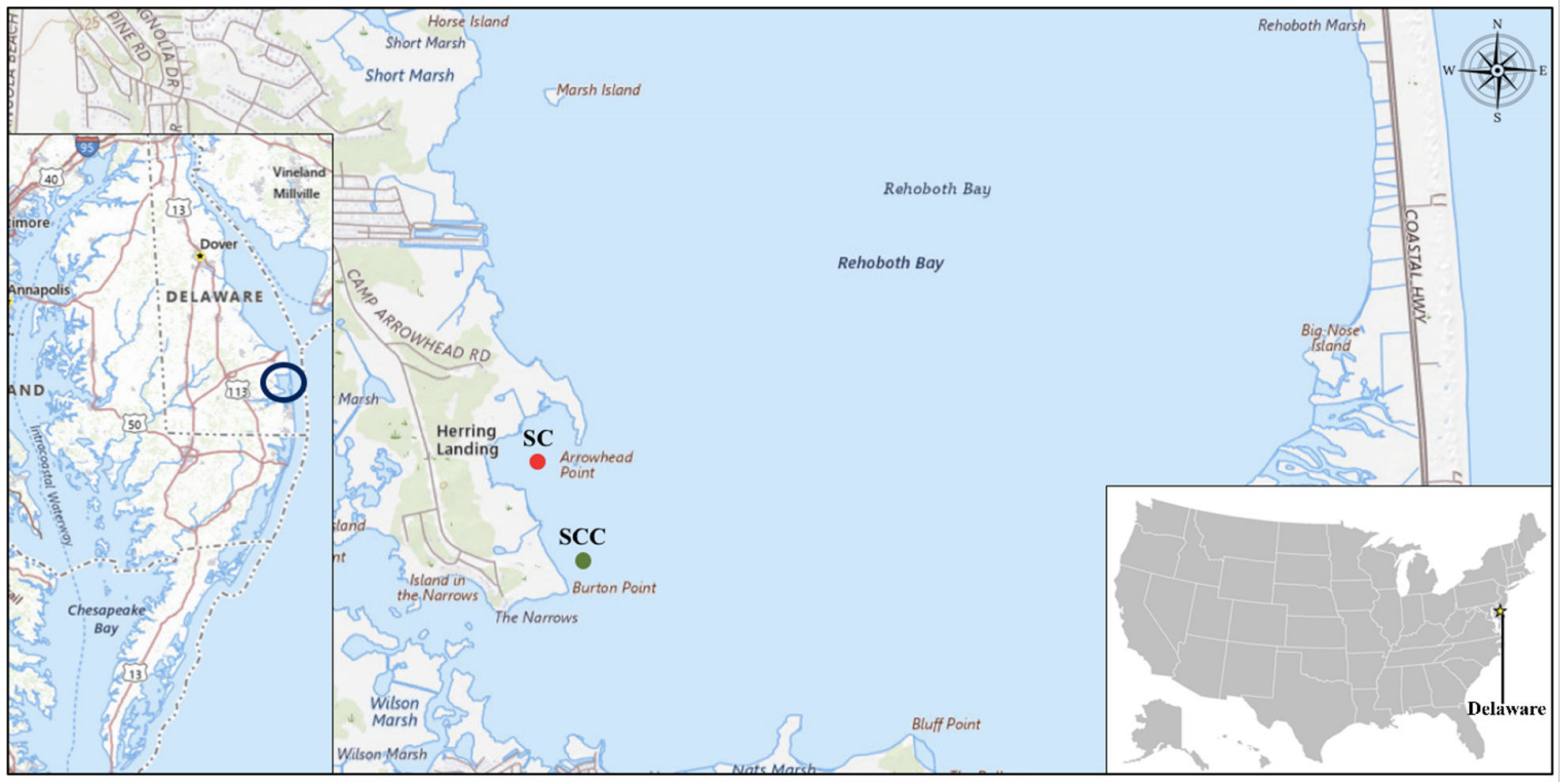
Geographic location of the study sites. This map shows the location of Sally Cove (SC) and its control site (SCC) within Rehoboth Bay in Delaware.

Sally Cove [38˚ 38.932′′ N, 075˚ 07.631′′ W] is a small beach and salt marsh located within Rehoboth Bay, Delaware (Fig. 10). The site is an oyster aquaculture farm that produces oysters from off-bottom and bottom cultures. In the off-bottom cultures, oysters are kept in floating cages suspended at 0.10 m in the seawater, whereas for the bottom cultures, the cages are submerged at depths ranging from approximately 1.60 to 1.80 m in the seawater. A control site [38˚ 38.613′′ N, 75˚ 07.575′′ W] within Sally Cove that is without oyster cultures was included in this study. Rehoboth Bay is one of the Delaware Inland Bays, alongside the Indian River and Little Assawoman Bays. Rehoboth Bay is very shallow and has poor and slow flushing rates (as high as 80–100 days), which makes it very sensitive to environmental changes (27).

Six liters of seawater and 12 oysters each from the off-bottom and bottom cultures at Sally Cove and 6 L each of off-bottom and bottom seawater from the control site were collected once per month from July to October 2023 using a water depth sampler (YSI Incorporated Xylem Analytics, Yellow Spring, OH, USA). Water quality parameters, including temperature (°C), salinity (ppt), pH, and dissolved oxygen (mg/L), were recorded *in situ* at the study sites using a ProDSS multiprobe instrument (YSI Incorporated Xylem Analytics, Yellow Spring, OH, USA). Oyster samples were bagged, and seawater samples were kept in 8 L amber HDPE bottles (Thermo Fisher Scientific, Waltham, MA, USA). Subsequently, the samples were subdivided into three groups of biological triplicates (three sets of 2 L seawater and three sets of 4 oysters) and kept in an insulated cooler with dry ice packs to maintain a temperature between 2 and 7°C. The samples were then transported to the One Health lab at Delaware State University within 24 h for processing.

### Processing of seawater and oyster samples

The seawater samples were centrifuged at 4,000 × *g* for 15 min at 25°C in a floor model centrifuge (Thermo Fisher Scientific, Waltham, MA, USA). The supernatants were discarded, and about 30 mL of sediment-concentrated seawater containing environmental DNA (eDNA) was kept for further analysis. The oyster samples were washed with distilled water, cleaned with a scrub brush, and then aseptically shucked using a sterile knife. The oyster tissues and liquors were homogenized in an Oster 16-speed Osterizer blender (Oster Manufacturing Company, Owosso, MI, USA) at high speed for 1 min to obtain oyster homogenates.

### Analysis of samples for the detection of *V. parahaemolyticus*

The samples were prepared for the detection of *V. parahaemolyticus,* as described by Elbashir et al. (46) with minor modifications. Twenty-five milliliters of sediment-concentrated seawater and 25 g of oyster homogenates were pre-enriched with 225 mL of alkaline peptone water (APW, Oxoid International Ltd, Basingstoke, UK) and 10 g of NaCl (Thermo Fisher Scientific, Waltham, MA) and incubated aerobically at 37°C for 24 h. From the pre-enriched seawater and oyster cultures, a 10 µL loop was streaked on thiosulfate-citrate bile salts-sucrose (TCBS) agar (Thermo Fisher Scientific, Waltham, MA, USA) and incubated aerobically at 37°C for 24 h. Five green/bluish-green presumptive colonies of *V. parahaemolyticus* on the TCBS agar were suspended in 100 µL of tryptone soya broth (TSB, Oxoid International Ltd, Basingstoke, UK) and incubated aerobically at 37°C for 24 h for the molecular detection of *V. parahaemolyticus*.

### Analysis of samples for the detection of Shiga-toxin-producing *E. coli* (STEC)

The samples were prepared for the detection of STEC following methods described by Al Qabili et al. (14) with minor modifications. Twenty-five milliliters of sediment-concentrated seawater and 25 g of oyster homogenates were pre-enriched with 225 mL of peptone water (PW, Oxoid International Ltd, Basingstoke, UK) and incubated at 37°C for 24 h. From the pre-enriched seawater and oyster cultures, a 10 µL loop was streaked on Sorbitol MacConkey (SMAC) agar (Alpha Biosciences, Baltimore, MD, USA) and incubated at 37°C for 24 h. Five colorless/pale presumptive colonies of STEC on the SMAC agar were suspended in 100 µL of Luria-Bertani broth (LB broth, Thermo Fisher Scientific, Waltham, MA, USA) and incubated at 37°C for 24 h for the molecular detection of STEC.

### Analysis of samples for the detection of *S. enterica* and *Shigella* spp.

The samples were prepared for the detection of *S. enterica* and *Shigella* spp. using methods described by Lattos et al. (69) and Wang et al. (70), respectively, with some modifications. Twenty-five milliliters of sediment-concentrated seawater and 25 g of oyster homogenates were pre-enriched with 225 mL of buffered peptone water (BPW, Alpha Biosciences, Baltimore, MD, USA) and incubated with shaking at 37°C for 24 h. From the pre-enriched seawater and oyster cultures, a 10 µL loop was streaked on xylose-lysine-deoxycholate (XLD) agar (Alpha Biosciences, Baltimore, MD, USA) and incubated at 37°C for 24 h. Five black presumptive colonies of *S. enterica* and five reddish pink presumptive colonies of *Shigella* spp. on the XLD agar were suspended in 100 µL of LB broth and incubated at 37°C for 24 h for the molecular detection of *S. enterica* and *Shigella* spp.

### Analysis of samples for the detection of *S. aureus*

The samples were prepared for the detection of *S. aureus* as described by Ouédraogo et al. (71) with minor modifications. Twenty-five milliliters of sediment-concentrated seawater and 25 g of oyster homogenates were pre-enriched with 225 mL of brain heart infusion (BHI) broth (Thermo Fisher Scientific, Waltham, MA, USA) and incubated at 37°C for 48 h. From the pre-enriched seawater and oyster cultures, a 10 µL loop was streaked on mannitol salt agar (MSA, Alpha Biosciences, Baltimore, MD, USA) and incubated at 37°C for 24 h. Five yellow presumptive colonies of *S. aureus* on the MSA were suspended in 100 µL of TSB and incubated at 37°C for 24 h for the molecular detection of *S. aureus*.

### Analysis of samples for the detection of *L. monocytogenes*

The samples were prepared for the detection of *L. monocytogenes* as described by Peratikos et al. (72). Briefly, 25 mL of sediment-concentrated seawater and 25 g of oyster homogenates were pre-enriched with 225 mL of Fraser broth (FB, Oxoid International Ltd, Basingstoke, UK) and incubated at 37°C for 48 h. From the pre-enriched seawater and oyster cultures, a 10 µL loop was streaked on agar *Listeria* according to Ottaviani Agosti (ALOA, Oxoid International Ltd, Basingstoke, UK) and incubated at 37°C for 24 h. Five cyan-green presumptive colonies of *L. monocytogenes* on the ALOA were suspended in 100 µL of TSB and incubated at 37°C for 24 h for the molecular detection of *L. monocytogenes*.

### Analysis of samples for the detection of *P. aeruginosa*

The samples were prepared for the detection of *P. aeruginosa* following methods described by Shahrokhi et al. (73). In brief, 25 mL of sediment-concentrated seawater and 25 g of oyster homogenates were pre-enriched with 225 mL of peptone water and incubated at 37°C for 48 h. From the pre-enriched seawater and oyster cultures, a 10 µL loop was streaked on cetrimide agar (PCA, Oxoid International Ltd, Basingstoke, UK) and incubated at 37°C for 24 h. Five blue-green presumptive colonies of *P. aeruginosa* on the PCA were suspended in 100 µL of TSB and incubated at 37°C for 24 h for the molecular detection of *P. aeruginosa*.

### Analysis of samples for the detection of *Clostridium* spp

The samples were prepared for the detection of *Clostridium* spp. using methods described by Li et al. (64) with some modifications. Briefly, 25 mL of sediment-concentrated seawater and 25 g of oyster homogenates were pre-enriched with 225 mL thioglycollate broth (FTG, Thermo Fisher Scientific, Waltham, MA, USA) and incubated anaerobically at 42°C for 48 h. From the pre-enriched seawater and oyster cultures, a 10 µL loop was streaked on reinforced clostridial agar (RCA, Thermo Fisher Scientific, Waltham, MA, USA) and incubated anaerobically at 42°C for 24 h. Five colorless/pale presumptive colonies of *Clostridium* spp. on the RCA were suspended in 100 µL of TSB and incubated anaerobically at 42°C for 24 h for the molecular detection of *Clostridium* spp.

### Molecular detection of bacteria using PCR and qPCR

Bacterial plasmids from suspensions were prepared for PCR and quantitative PCR (qPCR) using methods described by Jurinović et al. (74) with minor modifications. Briefly, the bacterial suspensions were centrifuged at 10,000 × *g* for 2 min in a microcentrifuge (Axygen Axyspin Refrigerated Microcentrifuge, Corning Life Sciences, Tewksbury, MA, USA). The supernatants were discarded, and the pellets were washed three times and resuspended in 100 µL of ultrapure nuclease-free water (Thermo Fisher Scientific, Waltham, MA, US). The resuspended pellets were boiled at 90°C for 15 min on a hot plate (Thermo Fisher Scientific, Waltham, MA, USA) and used as templates for PCR and qPCR.

Molecular confirmation of the bacteria was achieved using PCR and qPCR. The PCR reaction mixture in a 25 µL volume consisted of 12.5 µL of Go Taq Green G2 master mix (Promega Corporation, Madison, WI, USA), 0.5 µL each of 10 nM forward and reverse primers (Integrated DNA Technologies, Skokie, IL, USA), 2.5 µL of DNA template, and 9.0 µL of ultrapure nuclease-free water. The primers and amplification conditions for the targeted bacteria genes are shown in Table 2. Negative and positive control reactions were, respectively, set up using ultrapure nuclease-free water and cell lysate from pure bacterial cultures (Carolina Biological Supply, Burlington, NC, USA) as DNA templates. PCR reactions were performed in a Bio-Rad T100 thermal cycler (Bio-Rad, Hercules, CA, USA), and the products were visualized in a 1.5% agarose gel (Thermo Fisher Scientific, Waltham, MA, US) using a gel documentation system (Syngene-G Box, Frederick, MD, USA).

**TABLE 2:** PCR conditions and primer sequences used in this study

Bacteria	Targeted gene	Primer sequence (5′−3′)	Cycling conditions (35 cycles)	Product size (bp)	Reference
*V. parahaemolyticus*	*tlh*	F: ACTCAACACAAGAAGAGATCGACAA	Denaturation for 30 s at 95°C Annealing for 1 min at 60°C Extension for 1 min at 72°C	207	(75)
		R: GATGAGCGGTTGATGTCCAA			
Shiga-toxin-producing *E. coli*	*stx_2_*	F: GGGCAGTTATTTTGCTGTGGA	Denaturation for 30 s at 94°C Annealing for 30 s at 60°C Extension for 1 min at 72°C	131	(76)
		R: GAAAGTATTTGTTGCCGTATTAACGA			
*S. enterica*	*inv A*	F: TATCGCCACGTTCGGGCAA	Denaturation for 30 s at 94°C Annealing for 30 s at 50°C Extension for 35 s at 72°C	300	(77)
		R: TCGCACCGTCAAAGGAACC			
*Shigella* spp.	*ipaH*	F: CCTTGACCGCCTTTCCGATAC	Denaturation for 1 min at 94°C Annealing for 1 min at 59°C Extension for 1 min at 72°C	600	(78)
		R: CAGCCACCCTCTGAGAGTACTC			
*S. aureus*	*nuc A*	F: GCGATTGATGGTGATACGGTT	Denaturation for 1 min at 94°C Annealing for 30 s at 55°C Extension for 90 s at 72°C	270	(79)
		R: AGCCAAGCCTTGACGAACTAAAGC			
*L. monocytogenes*	*16S RNA*	F: CACGTGCTACAATGGATAG	Denaturation for 30 s at 94°C Annealing for 45 s at 50°C Extension for 45 s at 72°C	70	(80)
		R: AGAATAGTTTTATGGGATTA			
*P. aeruginosa*	*UCBP PPA14_00095* (*group_98983*)	F: CTCCGTGGAAAAGCAGTTG	Denaturation for 30 s at 95°C Annealing for 30 s at 58°C Extension for 30 s at 72°C	169	(81)
		R: GCGTATGCCGACGTAGAAT			
*Clostridium* spp.	*16S rRNA*	F: AAAGGAAGATTAATACCGCATAA	Denaturation for 30 s at 94°C Annealing for 1 min at 60°C Extension for 2 min at 68°C	722	(82)
		R: ATCTTGCGACCGTACTCCCC			

The qPCR reaction in a 25 µL volume in a 96-well plate consisted of 12.5 µL of SYBR Green PCR master mix (Thermo Fisher Scientific, Waltham, MA, US), 0.5 µL each of 10 nM forward and reverse primers, 2 µL of DNA template, and 9.5 µL of ultrapure nuclease-free water. The primers used to amplify the targeted bacteria genes are shown in Table 2. Negative and positive control reactions were, respectively, set up using ultrapure nuclease-free water and cell lysate from pure bacterial cultures, as DNA templates. The qPCR reactions were performed in an Applied Biosystems 7500 Real-time PCR system (Thermo Fisher Scientific, Carlsbad, CA, USA) with an optimized presence/absence cycle program as follows: 95°C for 10 min, followed by 40/50 cycles of 95°C for 15 s, 60°C for 1 min, and 72°C for 30 s. The reaction produced an amplification plot based on the presence or absence of the targeted bacteria genes.

## Data Availability

Data from this study are available upon request.

## SUPPLEMENTAL MATERIAL

Table S1 spectrum.03054-24-s0001.pdf Summary of the presence/absence and persistence of targeted bacteria in samples in this study.

ASM does not own the copyrights to Supplemental Material that may be linked to, or accessed through, an article. The authors have granted ASM a non-exclusive, world-wide license to publish the Supplemental Material files. Please contact the corresponding author directly for reuse.
