# Supplementary material for: Investigation of the presence and persistence of bacteria in seawater and oysters from an aquaculture farm in Rehoboth Bay, Delaware
Source: Microbiol Spectr. 2025 Apr 10;13(5):e03054-24. doi: 10.1128/spectrum.03054-24 (PMC12054098; doi:10.1128/spectrum.03054-24)
Supplement: Table S1 — Summary of the presence/absence and persistence of targeted bacteria in samples in this study. [file spectrum.03054-24-s0001.pdf]

TABLE S1 Summary of the presence and persistence of targeted bacteria in samples in this study.

Bacteria	Month	Samples					
		**OW-SC**	**BW-SC**	**OO-SC**	**BO-SC**	**OW-CS**	**BW-CS**
V. parahaemolyticus	July	+	+	+	+	+	+
	August	+	+	+	+	+	+
	September	+	+	+	+	+	+
	October	+	+	+	+	+	+
Shiga-toxin-producing *E. coli*	July	+	+	+	+	+	+
	August	+	+	+	+	+	+
	September	+	+	+	+	+	+
	October	+	+	+	+	+	+
S. enterica	July	+	+	+	+	+	+
	August	+	+	+	+	+	+
	September	+	+	+	+	+	+
	October	+	+	+	+	+	+
Shigella spp.	July	+	+	+	+	+	+
	August	+	+	+	+	+	+
	September	+	+	+	+	+	+
	October	–	+	–	+	–	+
S. aureus	July	+	+	+	+	+	+
	August	+	+	+	+	+	+
	September	+	+	+	+	+	+
	October	+	+	+	+	+	+
L. monocytogenes	July	+	+	+	+	+	+
	August	+	+	+	+	+	+
	September	+	+	+	+	+	+
	October	–	–	–	–	–	–
P. aeruginosa	July	+	+	+	+	+	+
	August	+	+	+	+	+	+
	September	+	+	+	+	+	+
	October	+	+	+	+	+	+
Clostridium spp.	July	+	+	+	+	+	+
	August	+	+	+	+	+	+
	September	+	+	+	+	+	+
	October	+	+	+	+	+	+

+ indicates the presence of the bacterium, and – indicates the absence of the bacterium. Abbreviations: OW-SC, Off-bottom seawater from Sally Cove; BW-SC, Bottom seawater from Sally Cove; OO-SC, Off-bottom-cultured oysters from Sally Cove; BO-SC, Bottom-cultured

oysters from Sally Cove; OW-CS, Off-bottom seawater from the control site; BW-CS, Bottom seawater from the control site.
